# The Use of Surrogate Endpoints in Regulating Medicines for Cardio-Renal Disease: Opinions of Stakeholders

**DOI:** 10.1371/journal.pone.0108722

**Published:** 2014-09-30

**Authors:** Bauke Schievink, Hiddo Lambers Heerspink, Hubert Leufkens, Dick De Zeeuw, Jarno Hoekman

**Affiliations:** 1 Department of Clinical Pharmacy and Pharmacology, University of Groningen, University Medical Center Groningen, Groningen, The Netherlands; 2 Utrecht Institute for Pharmaceutical Sciences, Division of Pharmacoepidemiology and Clinical Pharmacology, Utrecht University, Utrecht, The Netherlands; 3 Medicines Evaluation Board, Utrecht, The Netherlands; Moffitt Cancer Center, United States of America

## Abstract

**Aim:**

There is discussion whether medicines can be authorized on the market based on evidence from surrogate endpoints. We assessed opinions of different stakeholders on this topic.

**Methods:**

We conducted an online questionnaire that targeted various stakeholder groups (regulatory agencies, pharmaceutical industry, academia, relevant public sector organisations) and medical specialties (cardiology or nephrology vs. other). Participants were enrolled through purposeful sampling. We inquired for conditions under which surrogate endpoints can be used, the validity of various cardio-renal biomarkers and new approaches for biomarker use.

**Results:**

Participants agreed that surrogate endpoints can be used when the surrogate is scientifically valid (5-point Likert response format, mean score: 4.3, SD: 0.9) or when there is an unmet clinical need (mean score: 3.8, SD: 1.2). Industry participants agreed to a greater extent than regulators and academics. However, out of four proposed surrogates (blood pressure (BP), HbA1c, albuminuria, CRP) for cardiovascular outcomes or end-stage renal disease, only use of BP for cardiovascular outcomes was deemed moderately accurate (mean: 3.6, SD: 1.1). Specialists in cardiology or nephrology tended to be more positive about the use of surrogate endpoints.

**Conclusion:**

Stakeholders in drug development do not oppose to the use of surrogate endpoints in drug marketing authorization, but most surrogates are not considered valid. To solve this impasse, increased efforts are required to validate surrogate endpoints and to explore alternative ways to use them.

## Introduction

Cardiovascular and renal disease place an increasing burden on the healthcare system because of a growing incidence of diabetes and a high unmet need in useful protective therapies. The use of surrogate endpoints in clinical trials reduces the time to marketing authorization, which provides patients with earlier access to new medicines and lowers drug development costs [1]–[4]. However, there is a long-standing debate whether surrogate endpoints are valid proxies of clinically meaningful outcomes, especially in the prevention of cardiovascular and renal disease [5]–[9]. The debate has recently been reinvigorated by results from clinical trials that showed promising effects of medicines on surrogate endpoints without any effect on clinically meaningful outcomes [9]–[11]. For example, the anti-diabetic medicine rosiglitazone reduces the surrogate HbA1c, yet increases the risk of myocardial infarction [12], [13]; the antihypertensive medicine aliskiren increased the risk of stroke in the ALTITUDE trial despite reducing blood pressure and albuminuria [14], [15], and sibutramine increases risk of myocardial infarction and stroke despite lowering body weight [16].

Despite the debate, it remains unclear how stakeholders in drug development perceive the current use of surrogate endpoints in the marketing authorization of medicines. Therefore, we conducted a survey to assess opinions on the utility and validity of surrogate endpoints, with a focus on surrogates used for cardio-renal disease.

## Methods

### Ethics statement

We did not require IRB approval for conducting the presented survey, which is in compliance with the Dutch regulations on research with human participants. All gathered data was handled anonymously.

### Survey design

An online survey (see Survey Form S1) was designed with software from SurveyMonkey (www.surveymonkey.com, Palo Alto, CA, USA). The survey was checked for content validity by a pilot panel consisting of regulators from the Dutch Medicines Evaluation Board (MEB) and academic employees working at the University Medical Center Groningen. We targeted regulatory agencies (e.g. FDA, EMA), representatives from the pharmaceutical industry, relevant public sector organizations (e.g. Critical Path Institute (C-path), National Institute for Health and Care Excellence (NICE), National Institutes of Health (NIH)) and academic clinicians, including specialists in cardiology or nephrology as well as other specialists. The survey contained questions on the general use of surrogate endpoints, and on the validity of currently used surrogate endpoints for cardio-renal disease, and biomarkers that have been proposed as surrogates. We included blood pressure, HbA1c, albuminuria and CRP as surrogate endpoints for end-stage renal disease or cardiovascular (CV) disease (composite of myocardial infarction, stroke and CV death), while weight, carotid intima thickness and left ventricular hypertrophy were only included as surrogates for CV disease. We also included a medicine case scenario with questions on the use and validity of a composite score capturing the effect on multiple biomarkers as surrogate endpoint for clinically meaningful outcomes. Answers were provided on a 5-point Likert response format (i.e. strongly disagree, disagree, neutral, agree, strongly agree corresponding to a score of 1 to 5), ranking format or multiple choice format. We pre-specified to analyze differences in opinions between stakeholder groups and medical specialties.

### Sampling and population

Due to the relatively small and specialized population, we used purposeful sampling at stakeholder level to include participants. We did not perform a formal sample size calculation but strived to create equally sized stakeholder groups. The sample consisted of all participants from two international conferences on the topic of regulatory science and clinical trial design, where the use of surrogate endpoints was discussed. We observed a low participation rate of regulators and therefore invited additional participants from the European Medicines Agency (EMA) and assessors from the Dutch MEB. Participants were targeted by e-mail and a maximum of two reminders were sent in a time span of two months.

### Statistical analysis

Means and standard deviations (SD) were computed for questions based on a 5-point Likert response format. All reported p values were calculated by ANCOVA adjusted for age, gender, cardio-renal profession, stakeholder group and years of experience. Tukey HSD post-hoc tests were used for pairwise comparison between industry participants, regulators and academics. All other questions were analyzed non-parametrically. Participants from public sector organizations were excluded from comparisons between stakeholders due to small sample size. Background characteristics of participants that partially and completely filled out the survey were similar. Question answers were therefore analyzed with all available data. Analyses were conducted with R version 3.0.1 (R Foundation for Statistical Computing, Vienna, Austria. http://www.r-project.org).

## Results

### Survey and background characteristics

Background characteristics of surveyed participants are listed in Table 1. The population consisted of 193 individuals. A total of 74 persons participated (38% response): 18 representing the pharmaceutical industry, 18 from regulatory agencies, 34 from academia and 4 with other backgrounds, including public sector organizations (e.g. C-path, NICE, NIH). A total of 55 respondents (70%) were medical specialists in cardiology or nephrology. Median years of professional experience of all participants was 10 to 15 years. Most respondents were from the United States or Europe (91%), with a ratio of approximately 1∶1. No statistical differences between respondents and non-responders in the distribution of geographical location and gender were found.

**Table 1:** Characteristics of respondents.

Characteristic	Number (%)
Respondents	74 (38%*)
Males	53 (72.6%)
Age group (years)	
18–24	1 (1.4%)
25_–_34	2 (2.7%)
35–44	21 (28.4%)
45–54	26 (35.1%)
55–64	19 (25.7%)
64–75	5 (6.8%)
75+	0 (0%)
Experience	
0–5 years	9 (12.2%)
5–10 years	11 (14.9%)
10–15 years	21 (28.4%)
15+ years	33 (44.6%)
Stakeholders	
regulator	18 (24.3%)
industry	18 (24.3%)
academia	34 (46.0%)
other	4 (5.4%)
Specializations	
cardio-renal	55 (74.3%)
other	19 (25.7%)

*Percentage compared to surveyed population.

### Stakeholders

**Figure 1:**
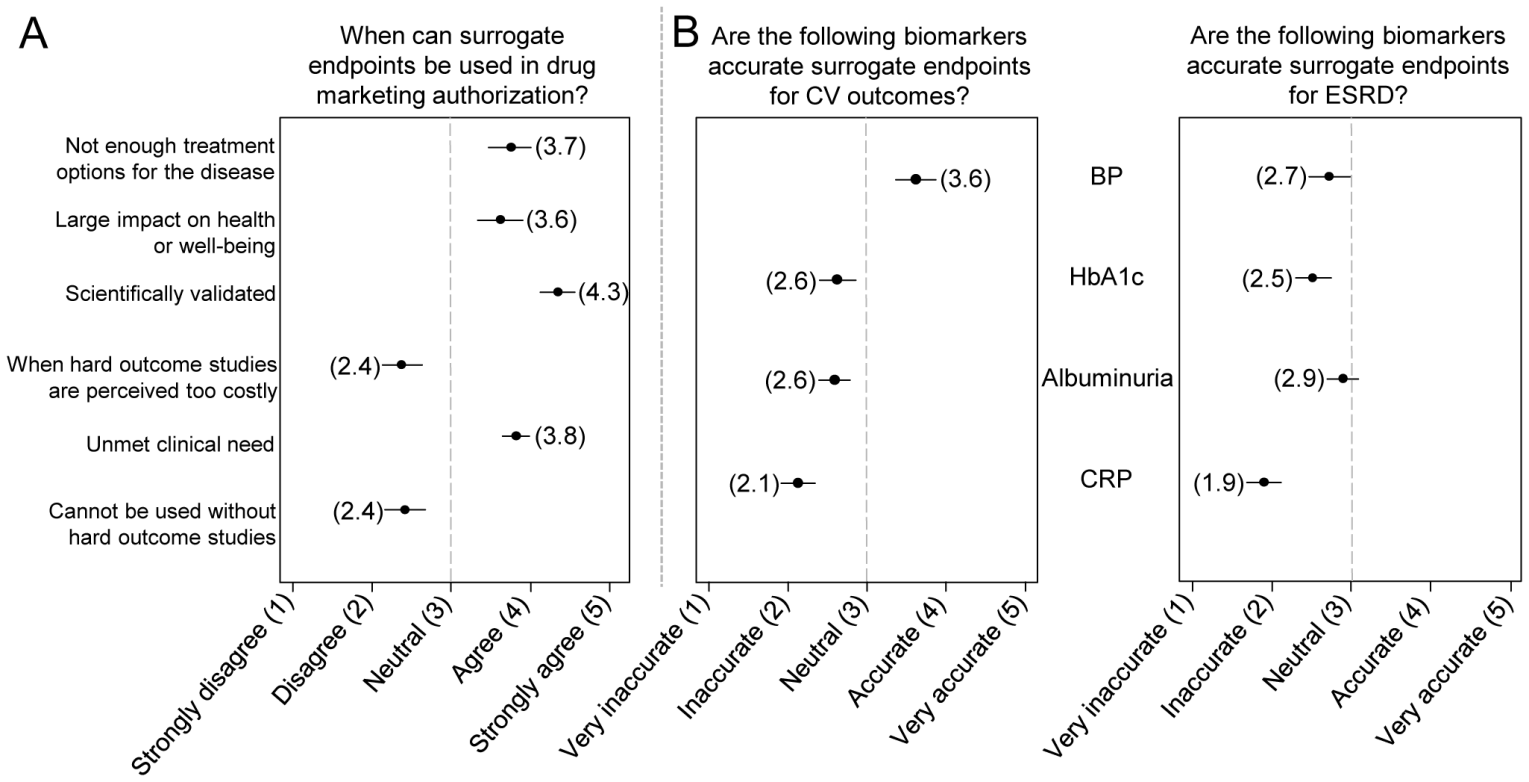
Pooled responses to survey questions. A: pooled responses (mean+95% CI) of all stakeholders on when surrogate endpoints can be used in drug marketing authorization, provided that a post-marketing study with hard outcomes is conducted. B: pooled answers on which biomarkers are perceived as accurate surrogate endpoints for either cardiovascular outcomes or end-stage renal disease. Absolute mean values are provided in brackets. Abbreviations: CV, cardiovascular; ESRD, end-stage renal disease; BP, blood pressure; CRP, C-reactive protein.

As shown in Figure 1A, there was consensus among stakeholder groups that surrogate endpoints can be used in drug marketing authorization under certain conditions. Specifically, all stakeholder groups agreed that a scientifically valid surrogate endpoint (pooled mean: 4.3, SD: 0.9) or an unmet clinical need are valid conditions for surrogate endpoint use, provided that a post-marketing study with hard outcomes is conducted (pooled mean: 3.8, SD: 1.2).

Industry participants were more positive towards the use of surrogate endpoints than both regulators and academic clinicians (Table 2). Industry participants were also more positive towards the statement that surrogate endpoints can be used when hard clinical outcome studies are perceived as too costly (mean 3.4, SD: 0.9). Academic clinicians and regulators ranked surrogate endpoints as the most beneficial for the pharmaceutical industry, while industry participants ranked surrogate endpoints as the most beneficial for patients (P<0.001 for difference).

**Table 2:** Question: When can surrogate endpoints be used?

	Stakeholder groups				Medical specialty			Pooled mean
	Regulator	Academia	Industry	P	CR	Other	P	
Not enough treatment options	3.5 (1.2)	3.6 (1.2)	4.5 (0.6)	0.012	3.9 (1.1)	3.4 (1.3)	0.24	3.7 (1.2)
Large impact on health and well-being	3.3 (1.4)	3.4 (1.2)	4.6 (0.5)	0.001	3.8 (1.2)	3.2 (1.3)	0.08	3.6 (1.2)
Scientifically validated	4.3 (0.7)	4.3 (1.0)	4.8 (0.4)	0.058	4.4 (0.9)	4.2 (1.0)	0.63	4.3 (1.0)
Hard outcome studies are too costly	2.1 (0.7)	2.1 (1.0)	3.4 (0.9)	<0.001	2.4 (1.1)	2.4 (1.1)	0.86	2.4 (1.1)
Unmet clinical need	3.9 (1.1)	3.4 (1.1)	4.7 (0.6)	<0.001	3.8 (1.2)	3.7 (1.2)	0.96	3.8 (1.2)
*Cannot* be used without hard outcome studies	2.4 (1.0)	2.9 (1.1)	1.6 (0.6)	<0.001	2.4 (1.1)	2.5 (1.1)	0.55	2.4 (1.1)

Statements on when surrogate endpoints can be used in drug marketing authorization, provided that a post-marketing study with hard outcomes will be conducted. Results from a 5-point Likert response format (strongly disagree – strongly agree) are presented in mean (SD) according to stakeholder group or medical specialty group (cardio-renal vs. no cardio-renal). Statistical differences between groups are mainly driven by differences of industry respondents vs. regulators and academia. Abbreviations: CR, cardiorenal background; P, P value.

Despite the positive attitude towards the use of surrogate endpoints among all stakeholders, they did not consider most currently used surrogates such as blood pressure, HbA1c, albuminuria and CRP valid substitutes for end-stage renal disease and cardiovascular (CV) outcomes. Only blood pressure for CV outcomes was considered a moderately accurate surrogate endpoint (pooled mean: 3.6 SD: 1.1; Figure 1B). Industry valued the accuracy of biomarkers consistently higher (exception: HbA1c for CV outcomes, Table 3). Additionally, all stakeholder groups indicated that weight, carotid intima thickness and left ventricular hypertrophy are not valid and should not be qualified by regulators as surrogate endpoints for CV outcomes in drug marketing authorization (data not shown).

**Table 3:** Question: are these biomarkers accurate surrogates?

	Stakeholder groups				Medical specialty			Pooled mean
	Regulator	Academia	Industry	P	CR	Other	P	
BP for CV outcomes	3.3 (1.1)	3.7 (1.0)	4.1 (0.7)	0.07	3.8 (1.0)	3.1 (1.0)	0.03	3.6 (1.1)
BP for ESRD	2.6 (0.7)	2.8 (0.9)	2.9 (0.9)	0.67	2.8 (0.9)	2.6 (0.7)	0.79	2.7 (0.9)
HbA1c for CV outcomes	2.8 (0.9)	2.6 (1.0)	2.7 (0.8)	0.87	2.7 (1.0)	2.3 (0.6)	0.28	2.6 (0.9)
HbA1c for ESRD	2.5 (1.0)	2.5 (1.1)	2.7 (0.7)	0.78	2.6 (1.0)	2.1 (0.7)	0.13	2.5 (1.0)
Albuminuria for CV outcomes	2.3 (0.7)	2.7 (0.8)	2.8 (0.8)	0.18	3.1 (0.8)	2.7 (0.8)	0.07	2.6 (0.8)
Albuminuria for ESRD	2.9 (0.9)	2.8 (1.0)	3.4 (0.7)	0.07	3.0 (1.0)	2.7 (1.0)	0.48	2.9 (1.0
CRP for CV outcomes	1.8 (0.7)	2.2 (0.9)	2.5 (0.9)	0.06	2.1 (0.9)	2.3 (0.9)	0.17	2.1 (0.9)
CRP for ESRD	1.9 (0.7)	1.9 (0.9)	2.1 (0.8)	0.70	1.8 (0.8)	2.1 (0.7)	0.11	1.9 (0.8)

Qualification of four biomarkers as accurate surrogate endpoints for CV outcomes or ESRD. Results from a 5-point Likert response format (strongly disagree – strongly agree) are shown in mean+SD according to professional background or medical specialty. Abbreviations: BP, blood pressure; CV, cardiovascular; ESRD, end-stage renal disease; CRP, C-reactive protein; CR, cardio-renal background; P, P value.

### Cardio-renal specialty

Specialists in cardiology or nephrology tended to agree more to the proposed statements regarding valid conditions for surrogate endpoint use compared to participants that are active in other fields. However, none of these differences were statistically significant (Table 2). Respondents with a specialty in cardiology or nephrology perceived blood pressure (mean: 3.8 vs 3.1, P<0.05) for CV outcomes as more accurate than those in other fields. Significant differences for the validity of other biomarkers were not observed (Table 3).

### Use of a risk score based on multiple biomarkers

We presented a hypothetical case of an antihypertensive medicine that fulfilled all the regulatory requirements for marketing authorization, including a significant reduction in blood pressure compared to placebo (Textbox S1). We found that 41 respondents were willing to accept this particular medicine for marketing authorization, while 18 respondents indicated that the medicine could not be marketed before conducting studies with clinically meaningful outcomes. Willingness to accept the medicine decreased significantly after showing that a risk score incorporating medicine-induced changes in multiple biomarkers predicted no CV protective effect of the medicine. Respondents indicated that such predictions incorporating medicine-induced changes in multiple biomarkers may be particularly useful for the selection of promising medicine candidates by pharmaceutical companies in phase II studies (mean: 3.9, SD: 0.9). Industry respondents tended to be more positive towards the use of predictions based on multiple biomarkers than other respondents (Table 4).

**Table 4:** Statements on use of multiple biomarkers.

	Stakeholder groups				Profession			Pooled mean
	industry	regulator	academia	P	CR	other	P	
more accurate predictions on hard outcomes than a single biomarker	4.0 (0.6)	3.9 (0.6)	3.3 (0.8)	0.01	3.6 (0.8)	3.3 (1.1)	0.39	3.5 (0.8)
select promising drug candidates during phase II clinical trials	4.5 (0.7)	3.8 (1.0)	3.9 (0.6)	0.01	4.0 (0.7)	3.5 (1.3)	0.16	3.9 (0.9)
substitute for hard clinical outcome studies, post-marketing studies required	3.5 (1.3)	3.1 (0.9)	2.7 (1.1)	0.11	2.9 (1.2)	2.8 (1.2)	0.90	2.9 (1.2)
substitute for hard clinical outcome studies, no post-marketing studies	2.2 (1.1)	2.3 (0.9)	1.6 (0.6)	0.02	1.9 (0.8)	1.8 (0.8)	0.93	1.9 (0.8)
no benefit for current registration practice	2.1 (0.9)	2.5 (0.9)	2.8 (0.9)	0.09	2.6 (1.0)	2.3 (0.8)	0.51	2.5 (0.9)
if multiple markers affect risk, approval cannot be based on a single marker	4.0 (0.8)	3.8 (0.7)	3.9 (0.8)	0.76	3.8 (0.9)	3.7 (0.6)	0.50	3.8 (0.8)
regulators should stimulate development of tools using multiple biomarkers	4.2 (0.6)	3.5 (1.0)	3.7 (1.0)	0.16	3.8 (1.0)	3.6 (0.8)	0.33	3.8 (1.0)

Statements on use of multiple biomarkers instead of single biomarkers to assess drug effects. Results from a 5-point Likert response format (strongly disagree – strongly agree) are shown in mean+SD according to professional background or medical specialty. Significant P values are driven by industry vs. academia (statement 1 and 2) and regulators vs. academia (statement 4). Abbreviations: CR, cardiorenal background.

## Discussion

We conducted an online survey to assess the opinions of stakeholders on the use of surrogate endpoints in marketing authorization of medicines. Although respondents generally agreed that there are valid reasons for use of surrogate endpoints, they did not perceive currently accepted surrogates as well as novel surrogates for cardiovascular and renal endpoints as valid.

Our results indicate an impasse in the perception and use of surrogate endpoints. In the past, surrogate endpoints such as blood pressure, cholesterol and glucose metabolism have been endorsed by regulators and used to allow medicines on the market, after which the effect on clinically meaningful outcomes was established in the post-marketing phase. However in recent years, many medicines authorized on the base of surrogate endpoints were shown to be harmful after results from trials with clinical meaningful outcomes became available. As a result, there is fierce debate among stakeholders about the use and purpose of current surrogate endpoints in the marketing authorization of medicines. Against this background, we foresee three ways along which the future use of surrogate endpoints in regulating medicines may evolve.

Firstly, reducing reliance on surrogate endpoints in marketing authorization of medicines may be a desired approach in light of recent experiences. Indeed, a call for less reliance on surrogate endpoints has been regularly expressed in the academic community [5], [8], [9], [17]. Use of surrogate endpoints could be restricted to situations where measuring clinically meaningful outcomes is not feasible, either due to practical or ethical concerns. For example, surrogates could be used in situations where it takes too long to measure a clinically meaningful endpoint due to slow disease progression [17]. This approach does not necessarily imply that the use of surrogate endpoints should be completely abandoned as evidence from biomarkers may provide important ancillary information in the regulatory assessment of the benefit and risks of medicines.

Less reliance on surrogate endpoints may also be achieved by requesting the conduct of hard clinical outcome trials. These trials could already be ongoing upon marketing authorization or be initiated shortly after medicines have been authorized based on surrogate endpoints. For instance, the FDA recently revised its guidelines on HbA1c-lowering medicines after the rosiglitazone incident by requiring hard clinical outcome studies to rule out harmful cardiovascular effects [18], thereby reducing reliance on HbA1c. Additionally, there is discussion whether regulatory authorities will require evidence on hard clinical outcomes for marketing authorization of upcoming PCSK9 inhibitors; novel medicine candidates that lower LDL cholesterol. As a result, several pharmaceutical companies have already initiated long-term hard outcome studies for PCSK9 inhibitors before marketing authorization [19], [20], [21], while other companies seem to base their drug development programs on the premise that LDL cholesterol is still considered a valid surrogate endpoint, knowing that a hard-clinical outcome study may be requested by regulatory authorities as a post-marketing commitment [22].

Secondly, further validation efforts may be conducted to rigorously evaluate currently used and proposed surrogate endpoints. Several criteria for formal scientific validation of surrogate endpoints have been proposed. Most of them require that there is thorough scientific understanding of the mechanistic relation between the surrogate and the hard clinical outcome as well as extensive preclinical (including animal studies) and clinical evidence confirming a quantifiable relationship between (treatment-induced) change in the surrogate outcome and change in the true clinical outcome [23]. One of the most well-known criteria for validation of surrogate endpoints was given by Prentice, who provided four operational criteria for the use of surrogates in clinical trials to ‘capture any relationship between the treatment and the true endpoint’ [24]. While the Prentice criteria are widely regarded as principles to scientifically validate a biomarker as surrogate endpoint, there is discussion whether they can reasonably be implemented in practice. In response, some scientists have proposed to validate surrogate endpoints based on the proportion of treatment effect explained or the strength of the relationship with the outcome of interest, as a quantifiable and less stringent measure [25], [26]. However, there is no golden rule on how much treatment effect needs to be explained before a biomarker qualifies.

Although currently no single accepted framework for the scientific validation of surrogate endpoints exists, there is widespread agreement that the conduct of prospective studies on clinically meaningful outcomes as well as retrospective analysis on data from already conducted clinical trials are key to further evaluate used and proposed surrogate endpoints. Prospective validation can be done in clinical trials by stratifying and randomizing a patient population based on their response on a surrogate marker [27]. This approach is currently used in the SONAR trial (Clinical Trial identifier NCT01858532) in which approximately 4,000 patients with type 2 diabetes and nephropathy are randomized to the investigational medicine or placebo as either responders or non-responders based on medicine-induced responses to the biomarker albuminuria. The biomarker-outcome association will be confirmed when the patient population randomized as responders also experience more benefit on clinically meaningful outcomes compared to non-responders.

Retrospective validation may rely on large scale meta-analyses of clinical trials and post-hoc analysis of individual patient data by measuring the relationship between short-term medicine-induced changes in biomarkers and medicine effects on clinically meaningful outcomes. In order to perform these analyses, incentives to make clinical trial data or clinical study reports accessible to academic investigators are needed [28]. Moreover, when relying on published data one should be aware of publication bias which may inflate the perceived association between biomarker and outcome [29], [30].

Thirdly, alternative ways of using biomarkers in the regulatory assessment of medicines are currently considered. The FDA and EMA recently introduced a procedural framework for the qualification of novel biomarkers [31], [32]. The qualification procedure facilitates early dialogue between regulatory authorities, scientists and companies to delineate a specific fit-for-purpose use and to objectively evaluate whether performance standards are met and claims on fit-for-purpose are supported [33], [34], [35]. It seems that these efforts mainly focus on the use of biomarkers for safety rather than efficacy purposes. For example, a consortium of stakeholders including scientists and representatives from the pharmaceutical industry under the umbrella of the Critical Path Institute focuses exclusively on the development of biomarkers for early prediction of nephrotoxicity or hepatotoxicity [36], while in Europe, several initiatives for safety markers are currently ongoing as part of the Innovative Medicines Initiative [37].

Another new approach is to use medicine-induced changes in multiple biomarkers to predict effects on clinically meaningful outcomes in early stages. The rationale for this approach is that many medicines have effects on multiple biomarkers, with each of these biomarkers being associated with changes in clinically meaningful outcomes, either positively or negatively. A score that integrates medicine-induced responses to multiple biomarkers may therefore better capture the medicine effect on clinically meaningful outcomes than changes in single biomarkers alone. Indeed, we recently developed a risk score that accurately predicted the efficacy of angiotensin receptor blockers on cardiovascular and renal endpoints in a post-hoc analysis [38]. Moreover, the score was prospectively validated by predicting the treatment effect of aliskiren on hard clinical outcomes in the ALTITUDE trial before the trial was completed [39]. A similar model has been developed by Archimedes, which uses multiple parameters from existing clinical trial data to predict cardiovascular risk [40]. In our survey, respondents valued the use of these scores to provide early insights in medicine efficacy, although it was not considered a replacement for conducting studies on clinically meaningful outcomes.

Our study has limitations. Firstly, we used a non-random sample for enrolment. Gathering a random sample was not feasible due to the relatively small and specialized population. Secondly, 38% of the targeted population responded to our survey. However, internet-based surveys traditionally have a low response rate [41]–[43]. Thirdly, the contribution of the regulatory community is mainly based on European input, not necessarily representing other authorities. Fourthly, we did not include community physicians, payers or patient groups as stakeholder. These parties mainly play a role in the post-marketing domain but also increasingly contribute to the decision-making process before and during marketing authorization.

In conclusion, stakeholders in drug development do not oppose to the use of surrogate endpoints although they consider most surrogates inaccurate substitutes for clinically meaningful outcomes. To solve this impasse, increased efforts are required to validate surrogate endpoints and to explore alternative ways to use them.

## Supporting Information

Textbox S1 **Details of the hypothetical drug X, as presented in our drug case scenario.**
(DOC)

Survey Form S1 **The survey form that was sent electronically to all participants.**
(PDF)
