# Supplementary material for: The Use of Surrogate Endpoints in Regulating Medicines for Cardio-Renal Disease: Opinions of Stakeholders
Source: PLoS One. 2014 Sep 30;9(9):e108722. doi: 10.1371/journal.pone.0108722 (PMC4182561; doi:10.1371/journal.pone.0108722)
Supplement: Textbox S1 — Details of the hypothetical drug X, as presented in our drug case scenario. (DOC) [file pone.0108722.s001.doc]

| **Introduction**  In the medicine case scenario we provided participants with information on the safety and efficacy of the hypothetical anti-hypertensive medicine X. Based on two pieces of information that were successively presented, participants were asked to evaluate whether they deemed medicine X was acceptable for marketing authorization.  **Setting 1:**  Participants were asked if they deemed medicine X acceptable for drug marketing authorization after showing them the medicine-induced biomarker responses provided in Table A. We specified that medicine X had a comparable safety and tolerability profile as other registered anti-hypertensive medicines and that all other regulatory requirements for medicine X were met (see supplemental Survey Form S1).  Table A: Average biomarker responses of medicine X versus placebo in two registration trials   | Biomarker | Medicine X | Placebo | | --- | --- | --- | | Systolic blood pressure, mmHg | -9 | -2.5 | | Diastolic blood pressure, mmHg | -4 | -0.5 | | HbA1c, % | -0.3 | -0.1 | | Hemoglobin, g/dl | -0.8 | -0.1 | | Potassium, mmol/L | +0.6 | -0.2 | | Uric acid, mmol/L | +0.4 | -0.4 | | Albumine:creatinine ratio, % | -6 | -4 | | Total cholesterol, mmol/L | -0.1 | -0.1 |   **Setting 2:**  Participants subsequently received additional information showing the predicted long-term efficacy of medicine X on cardiovascular outcomes, as provided in Table B. They were asked again whether they deemed the medicine acceptable for marketing authorization.  Table B:   | Predictions | Change in cardiovascular risk (versus placebo) | | --- | --- | | based on blood pressure-lowering effect | 15% reduction (p<0.01, 95% CI: -24% to -9%) | | based on changes in all biomarkers (Table A) | 4% increase (p=0.10, 95% CI: -3% to +11%) |   **Results:**  After presenting the information in setting 1, 41 respondents indicated that medicine X could be authorized on the market. 32 out of 41 respondents considered a post-marketing study (PMS) on hard clinical outcomes necessary, while 9 respondents indicated that a PMS was not necessary. Eighteen respondents indicated that medicine X could not be authorized on the market before conducting a hard outcome study. After presenting the information in setting 2, the number of respondents indicating that medicine X could be authorized on the market decreased from 41 to 27 (of which 24 considered a PMS necessary and 3 did not). The number of respondents indicating that medicine X could not be authorized on the market without hard clinical outcome studies increased from 18 to 32. Differences in answers between setting 1 and setting 2 were statistically significant (P<0.001, McNemar test). |
| --- | --- | --- | --- | --- | --- | --- | --- | --- | --- | --- | --- | --- | --- | --- | --- | --- | --- | --- | --- | --- | --- | --- | --- | --- | --- | --- | --- | --- | --- | --- | --- | --- | --- |

Abbreviations: PMS, post-marketing studies; CI, confidence interval.
