# Supplementary material for: The Use of Surrogate Endpoints in Regulating Medicines for Cardio-Renal Disease: Opinions of Stakeholders
Source: PLoS One. 2014 Sep 30;9(9):e108722. doi: 10.1371/journal.pone.0108722 (PMC4182561; doi:10.1371/journal.pone.0108722)
Supplement: Survey Form S1 — The survey form that was sent electronically to all participants. (PDF) [file pone.0108722.s002.pdf]

Introduction

In this survey we are interested in your opinion on the current and future use of surrogate endpoints in the marketing authorization of drugs. There is much debate regarding the exact definition of terms such as biomarkers and surrogate endpoints. For the purpose of this survey, we have defined surrogate endpoints as biomarkers that function as accepted substitutes for hard clinical endpoints in clinical trials. These biomarkers are accepted as surrogate endpoints because there is regulatory consensus that the biomarker is on the direct causal pathway from disease to hard clinical outcome, as shown in the figure below.

The survey is part of an ongoing research project that is conducted under the umbrella of the Escher project of the non-profit organization TI-Pharma (T6-503). The survey has been developed by a research team from the Department of Clinical Pharmacology from the University Medical Center Groningen.

The survey contains 17 questions divided in multiple sections. All data will be treated anonymously.

Figure: ideal case of an intervention targeting a surrogate endpoint which is on the direct causal pathway from disease to hard clinical outcome

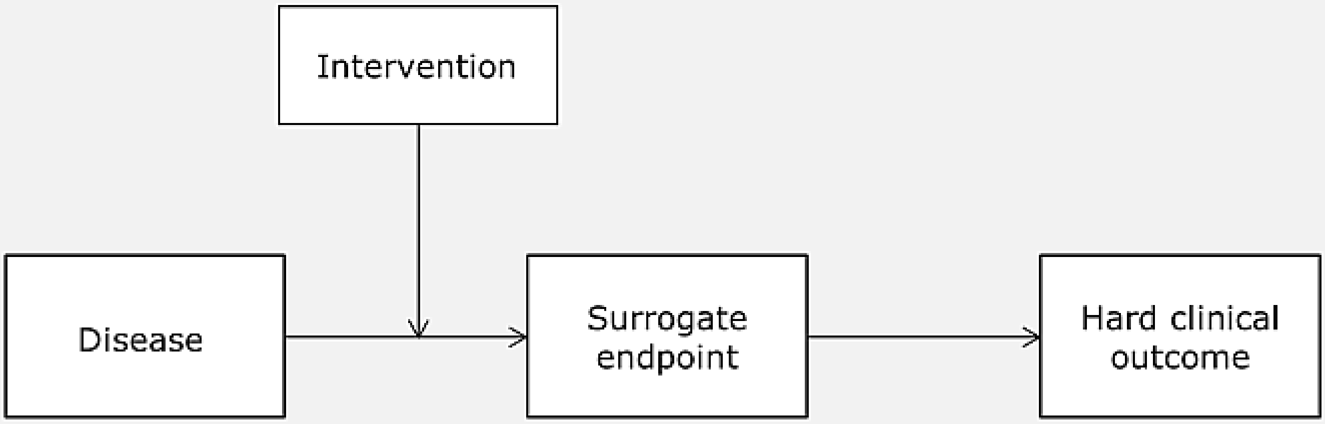

Background

First, we would like to know a bit more about your background and your general experience with the use of surrogate endpoints.

1. What is your gender?

- Female
- Male

2. What is your age?

- 18 to 24
- 25 to 34
- 35 to 44
- 45 to 54
- 55 to 64
- 65 to 74
- 75 or older

3. How long have you been professionally active in the pharmaceutical field?

- 0-5 years
- 5-10 years
- 10-15 years
- More than 15 years

4. What is your main professional background?

- I work in a regulatory institute (e.g. FDA, EMA)
- I work in a pharmaceutical company or CRO
- I work in a university or university hospital
- I work in a medical guideline organization
- I work in a patient support organization

Other (please specify)

5. Who is your main employer?

- ○ FDA
- ○ EMA, or a regulator on a national level within the EU
- ○ Other

6. What are your areas of specialization? (providing multiple answers is possible)

- ☐ Cardiology
- ☐ Nephrology
- ☐ Endocrinology
- ☐ Oncology
- ☐ Immunology
- ☐ Infectious Diseases
- ☐ Neurology

Other (please specify)

7. Have you been professionally involved in the development or marketing authorization of drugs targeted to the following surrogate endpoints?

	Yes	No
Blood pressure	☐	☐
Hba1c	☐	☐
Lipid (cholesterol)	☐	☐
CD4+ T-cell count	☐	☐
Tumour response/progression	☐	☐

Other (please indicate)

Current use of surrogate endpoints

We continue with questions on the use of surrogate endpoints in marketing authorization.

8. Drug registration based on surrogate endpoints might be beneficial for a wide range of stakeholders in the drug development process. In your opinion, who benefits most from the use of surrogate endpoints? Please use the dropdown menu to rank the groups from most benefit (1) to least benefit (4).

Note: answers change position while indicating your preferences.

- A: Patients
- B: Pharmaceutical companies
- C: Regulators
- D: Scientists

9. Please indicate when in your opinion surrogate endpoints could be used as substitutes for hard clinical endpoints in drug marketing authorization, provided that a post-marketing study with hard clinical endpoints will be conducted.

	Strongly disagree	Disagree	Neutral	Agree	Strongly agree
When there are not enough treatment options for the disease	◯	◯	◯	◯	◯
When the disease has a large impact on a patient's health and well-being	◯	◯	◯	◯	◯
When the surrogate endpoint is considered scientifically valid	◯	◯	◯	◯	◯
When the pharmaceutical industry claims it is too costly to use the hard clinical outcome in clinical trials	◯	◯	◯	◯	◯
When there is an unmet clinical need	◯	◯	◯	◯	◯
Drugs should not be marketed before a hard clinical outcome study is conducted	◯	◯	◯	◯	◯

Validity of surrogate endpoints in cardiovascular disease

In the remainder of the survey we are interested in your opinion on the use of surrogate endpoints in the cardiovascular field. The predictive accuracy of surrogate endpoints is being debated in this field due to a number of unexpected results from hard clinical outcome studies. A well-known example is rosiglitazone that due to its HbA1c-lowering effect was expected to decrease the risk of cardiovascular complications in diabetes patients. However, a post-marketing hard clinical outcome study revealed an increase in cardiovascular risk resulting in the withdrawal of the drug from the European market.

10. Please indicate whether you consider yourself knowledgeable to answer questions on the use of surrogate endpoints in the cardiovascular and/or diabetes field.

- ○ Yes
- ○ No

11. Please indicate whether you consider the following risk markers accurate surrogate endpoints for hard clinical outcomes in clinical trials.

	Very inaccurate	Inaccurate	Neutral	Accurate	Very accurate
Blood pressure-lowering to substitute for cardiovascular composite outcomes (stroke, MI, CV death)	◯	◯	◯	◯	◯
Blood pressure-lowering to substitute for End-Stage Renal Disease (ESRD)	◯	◯	◯	◯	◯
HbA1c-lowering to substitute for cardiovascular composite outcomes (stroke, MI, CV death)	◯	◯	◯	◯	◯
HbA1c-lowering to substitute for End-Stage Renal Disease (ESRD)	◯	◯	◯	◯	◯
HbA1c-lowering to substitute for retinopathy	◯	◯	◯	◯	◯
Albuminuria-lowering to substitute for cardiovascular composite outcomes (stroke, MI, CV death)	◯	◯	◯	◯	◯
Albuminuria-lowering to substitute for End-Stage Renal Disease (ESRD)	◯	◯	◯	◯	◯
C-reactive protein-lowering to substitute for cardiovascular composite outcomes (stroke, MI, CV death)	◯	◯	◯	◯	◯
C-reactive protein-lowering to substitute for End-Stage Renal Disease (ESRD)	◯	◯	◯	◯	◯

12. Given the current scientific evidence, should regulatory institutions accept the following proposed surrogate endpoints in drug marketing authorization?

	Not acceptable	Neutral	Acceptable
Use of albuminuria to subsitute for End Stage Renal Disease	◯	◯	◯
Use of albuminuria to substite for cardiovascular outcomes	◯	◯	◯
Use of C-reactive protein to substitute for cardiovascular outcomes	◯	◯	◯
Use of body weight to substitute for cardiovascular outcomes	◯	◯	◯
Use of Carotid Intima Thickness to substitute for cardiovascular outcomes	◯	◯	◯
Use of Left-Ventricular-Hypertrophy to substitute for cardiovascular outcomes	◯	◯	◯

Drug effects on multiple biomarkers - Background information for the remain...

In current registration practice, many cardiovascular drugs are approved based on efficacy on a single marker (e.g. blood pressure in case of an angiotensin II receptor blocker (ARB) for the treatment of hypertension), to which we will refer in this survey as the on-target marker. At the same time, these drugs may have an effect on other biomarkers to which we will refer in this survey as off-target markers. Such off-target markers can contribute either in a positive (e.g. albuminuria-lowering effect) way or a negative (e.g. hemoglobin-lowering effect) way to a drug's efficacy/safety profile. An example is visualized below, showing how the ARB Losartan has an effect on the on-target marker blood pressure and the off-target markers albuminuria, potassium and hemoglobin.

Predictions of long-term treatment effects may be different when taking into account changes in both on-target and off-target markers as compared to changes in the on-target marker alone. To provide insight in these differences, we have developed a novel risk score that predicts the effect of a drug on hard clinical outcomes based on changes in all measured on-target and off-target markers.

On the next page, a case will be presented that illustrates the use of the risk score.

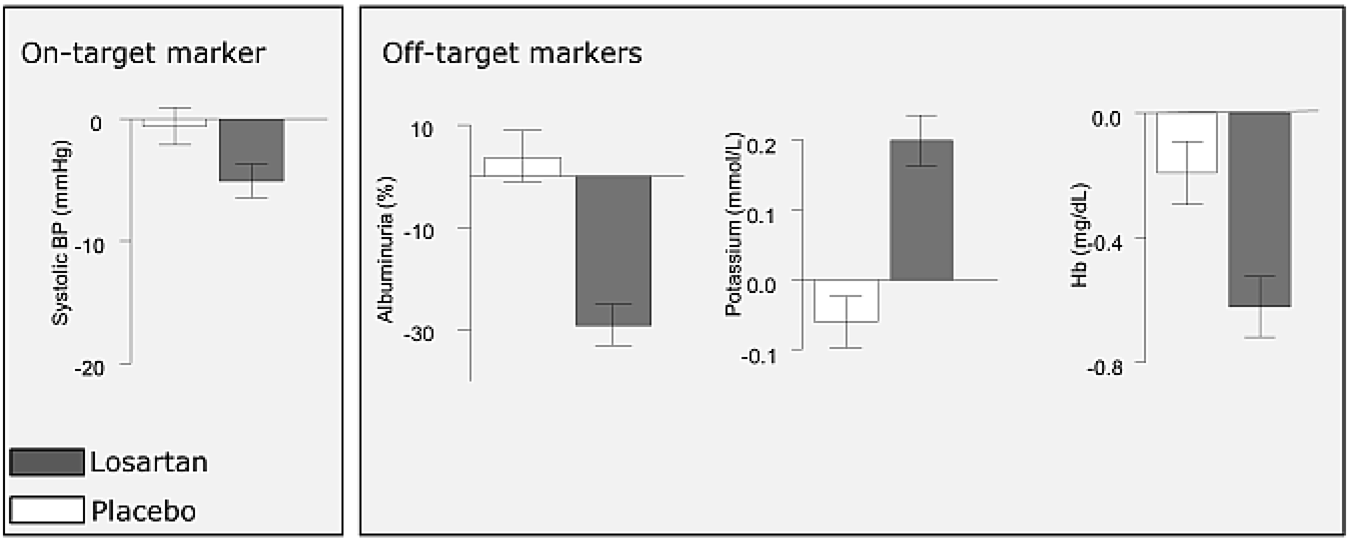

A drug application was submitted for a novel blood pressure-lowering agent called Drug X. Below you will find the most important details of the conducted clinical trials to establish safety and efficacy of Drug X.

The details of 2 registration studies for efficacy:
- Treatment: Drug X versus placebo
- Duration: 12 weeks
- Study population: 900 hypertensive patients drawn from the general population (450 in each group)
- Measurements: blood pressure and several additional markers

- A one year open-label study with drug X indicated no suspicion of a detrimental effect on cardiovascular hard clinical outcomes.

These studies showed that drug X was well tolerated and any side effects were similar to existing blood pressure-lowering agents.

The effects of drug X on multiple on and off-target markers are summarized in the table below.

Changes in markers after 12 weeks	Drug X	Placebo
Systolic blood pressure (mmHg)	-9	-2.5
Diastolic blood pressure (mmHg)	-4	-0.5
HbA1c (%)	-0.3	-0.1
Hemoglobin (g/dl)	-0.8	-0.1
Potassium (mmol/L)	+0.6	-0.2
Uric acid (mmol/L)	+0.4	-0.4
Albumine:creatinine ratio (%)	-6	-4
Total cholesterol (mmol/L)	-0.1	-0.1

13. Based on the information given above, should this drug be approved for market authorization?

- ◯ Yes, no post-marketing studies involving hard clinical endpoints are required
- ◯ Yes, but post-marketing studies involving hard clinical endpoints are necessary to validate the cardioprotective effect of Drug X
- ◯ No, follow-up studies with hard clinical endpoints are necessary before the drug can be registered

Given the observed changes in biomarkers, the following predictions were made concerning the treatment effect of Drug X on cardiovascular outcomes (stroke, MI and cardiovasular death), relative to placebo:

PREDICTIONS BASED ON BLOOD PRESSURE-LOWERING EFFECT:
15% reduction in cardiovascular risk
($p<0.01$, 95% CI: -24% to -9%)

PREDICTIONS WITH THE NEWLY DEVELOPED RISK SCORE BASED ON CHANGES IN ALL MEASURED BIOMARKERS:
4% increase in cardiovascular risk
($p=0.10$, 95% CI: -3% to +11%)

14. Based on the predictions given above, should this drug be approved for market authorization?

- ○ Yes, no post-marketing studies involving hard clinical endpoints are required
- ○ Yes, but post-marketing studies involving hard clinical endpoints are necessary to validate the cardioprotective effect of Drug X
- ○ No, follow-up studies with hard clinical endpoints are necessary before the drug can be registered

15. Suppose that the risk score is validated in prospective studies and is accurate in predicting the treatment effect of several drug classes in the cardiovascular field, please indicate to what extent you agree or disagree with the following statements on the use of the presented risk score in drug registration.

	Strongly disagree	Disagree	Neutral	Agree	Strongly agree
The score provides more accurate predictions on hard clinical outcomes than changes in single markers alone	○	○	○	○	○
The score may be used to select promising drug candidates during phase II clinical trials	○	○	○	○	○
Use of the score can substitute for hard clinical outcome studies, but post-marketing studies are required	○	○	○	○	○
Use of the score can substitute for hard clinical outcome studies, without further requirement of post-marketing studies	○	○	○	○	○
The score does not offer any benefit for current registration practice	○	○	○	○	○

16. Please indicate to what extent you agree or disagree with the following statements.

	Strongly disagree	Disagree	Neutral	Agree	Strongly agree
When the risk score predicts that changes in both on-target and off-target biomarkers affect cardiovascular risk, market approval cannot be based on changes in a single on-target marker alone	○	○	○	○	○
Regulators should stimulate the development of prediction tools that use multiple biomarkers	○	○	○	○	○

Thank you

You have completed the survey. We thank you for your cooperation. Should you have any further comments, please let us know by using the text box below.

17. Comments
